# 吉非替尼治疗32例肺腺癌脑转移的临床经验

**DOI:** 10.3779/j.issn.1009-3419.2015.09.05

**Published:** 2015-09-20

**Authors:** 建萍 许, 潇衍 刘, 晟 杨, 湘茹 张, 远凯 石

**Affiliations:** 100021 北京，北京协和医学院中国医学科学院肿瘤医院肿瘤内科 Department of Medical Oncology, Cancer Hospital Chinese Academy of Medical Sciences, Peking Union Medical College, Beijing 100021, China

**Keywords:** 吉非替尼, 肺肿瘤, 脑转移, Gefitinib, Lung neoplasms, Brain metastasis

## Abstract

**背景与目的:**

脑转移是晚期非小细胞肺癌（non-small cell lung cancer, NSCLC）常见的转移部位，预后欠佳。吉非替尼是一种表皮生长因子受体（epithelial growth factor receptor, EGFR）酪氨酸激酶抑制剂，用于治疗晚期NSCLC。本研究旨在探讨吉非替尼治疗肺腺癌脑转移的疗效及毒副反应。

**方法:**

回顾性分析32例肺腺癌脑转移患者的临床资料，所有患者均口服吉非替尼250 mg *Qd*，直到疾病进展或发生不可耐受的毒副反应。

**结果:**

全组32例患者的中位生存时间（median overall survival, mOS）和中位无进展生存时间（median progression-free survival, mPFS）分别为24.7个月和11.2个月，有效率（response rate, RR）和疾病控制率（disease control rate, DCR）分别为62.5%和93.8%。吉非替尼用于初治患者的mOS和mPFS分别为35.6个月和11.3个月，RR和DCR分别为75.0%和100.0%。吉非替尼用于复治患者的mOS和mPFS分别为18.6个月和6.7个月，RR和DCR分别为50.0%和83.3%。EGFR敏感性突变患者的mOS和mPFS分别为24.8个月和10.8个月，RR和DCR分别为75.0%和100.0%。*EGFR*突变状态不明患者的mOS和mPFS分别为35.6个月和12.3个月，RR和DCR分别为53.3%和86.7%。全组患者耐受性好，未观察到严重毒副反应。常见的毒副反应包括：皮疹15例（46.9%）、腹泻7例（21.9%）、口腔溃疡1例（3.1%）。

**结论:**

吉非替尼对肺腺癌脑转移患者有效率较高且耐受性好，可以作为肺腺癌脑转移患者的一种治疗选择。

## Abstract (en)

**Background and objective:**

Brain metastasis was frequent in non-small cell lung cancer (NSCLC) patients with poor prognosis. Gefitinib was an epidermal growth factor receptor (EGFR) tyrosine kinase inhibitor which has been used in the treatment of NSCLC. Our study was to evaluate the efficacy and toxicities of gefitinib in lung adenocarcinoma patients with brain metastases.

**Methods:**

We retrospectively reviewed clinical records of 32 lung adenocarcinoma patients with brain metastases, who had received gefitinib 250 mg *Qd* until disease progression or intolerable toxicities.

**Results:**

The median overall survival (mOS) and median progression-free survival (mPFS) were 24.7 months and 11.2 months, respectively. Response rate (RR) and disease control rate (DCR) were 62.5% and 93.8%, respectively. The mOS and mPFS of gefitinib-naive patients were 35.6 months and 11.3 months, respectively, and RR and DCR were 75.0% and 100.0%, respectively. The mOS and mPFS of gefitinib treatment patients were 18.6 months and 6.7 months, respectively, and RR and DCR were 50.0% and 83.3%, respectively. The mOS and mPFS of patients with sensitive *EGFR* mutation were 24.8 months and 10.8 months, respectively, and RR and DCR were 75.0% and 100.0%, respectively. The mOS and mPFS of patients with unknown EGFR status were 35.6 months and 12.3 months, respectively, and RR and DCR were 53.3% and 86.7%, respectively. Treatment was well tolerated and no severe toxicities were observed. Common toxicities include: rash in 15 patients (46.9%), diarrhea in 7 cases (21.9%) and oral ulcer in 1 case (3.1%).

**Conclusion:**

Gefitinib was highly effective and well tolerated in lung adenocarcinoma patients with brain metastases, and could be recommended as a treatment choice for this population.

脑转移是晚期非小细胞肺癌（non-small cell lung cancer, NSCLC）常见的转移部位，约10%-25%的患者诊断时已出现中枢神经系统转移，40%-50%的患者在治疗中出现中枢神经系统转移^[1, 2]^。近年来伴随治疗手段的进展，NSCLC患者的生存时间普遍延长，脑转移的发生率也随之增加。脑转移严重影响患者的生存时间和生活质量，出现脑转移后患者的中位生存时间为3个月-6个月，其中未治疗患者的中位生存时间仅4周-11周^[3]^。由于血脑屏障的存在，化疗药物在脑组织内的浓度较低，用于肺癌常见的化疗药物如紫杉类、铂类、培美曲塞、吉西他滨、拓扑替康等治疗脑转移灶的疗效欠佳^[4-6]^。

在肺癌中，表皮生长因子受体（epithelial growth factor receptor, *EGFR*）基因突变是驱动性基因突变，阻断突变的EGFR活化，可以有效抑制肿瘤细胞的增殖^[7, 8]^。研究^[9]^证实了EGFR小分子酪氨酸激酶抑制剂（EGFR tyrosine kinase inhibitor, EGFR-TKI）治疗NSCLC的有效性和安全性。吉非替尼等EGFR-TKI的问世，为NSCLC脑转移患者提供了新的选择。我们的研究旨在探讨吉非替尼治疗肺腺癌脑转移的疗效及毒副反应。

## 1 资料与方法

### 1.1 资料

2010年3月-2014年12月，32例脑转移的肺腺癌患者于中国医学科学院肿瘤医院接受吉非替尼（易瑞沙）治疗。其中男性13例，女性19例，中位年龄58岁（41岁-79岁）；既往吸烟者6例，不吸烟者26例；病理类型全部为肺腺癌，其中17例患者为*EGFR*敏感性突变（19或21外显子突变），15例患者*EGFR*突变状态不明。15例EGFR状态未明的患者中既往非吸烟者13例，女性11例，中位年龄59岁（45岁-73岁）。所有患者均在服用吉非替尼前确诊脑转移，其中21例患者接受颅脑放疗（WBRT/γ刀）。全组患者中，吉非替尼初治的患者为20例，复治患者12例，其中二线治疗的患者8例，三线及三线以上治疗的患者4例。

### 1.2 方法

#### 1.2.1 治疗方案

所有患者每天口服吉非替尼250 mg，直到病变进展或发生不可耐受的不良反应。

#### 1.2.2 评价标准

采用实体瘤疗效评价标准（Response Evaluation Criteria in Solid Tumors, RECIST）1.0评价疗效，分为完全缓解（complete response, CR）、部分缓解（partial response, PR）、稳定（stable disease, SD）以及疾病进展（progressive disease, PD），以（CR+PR）例数/总例数表示客观有效率（response rate, RR），以（CR+PR+SD）例数/总例数表示疾病控制率（disease control rate, DCR）。无进展生存时间（progression-free survival, PFS）定义为第一次服用易瑞沙的时间到第一次影像学上发现肿瘤进展的时间；总生存期（overall survival, OS）定义为第一次服用易瑞沙的时间到病例死亡时间或者最后一次随访时间。毒性反应依据美国国立癌症研究所通用不良反应术语标准（National Cancer Institute Common Toxicity Criteria, NCI CTC）4.0标准进行评价。

### 1.3 统计学方法

采用SPSS 22.0统计软件进行数据分析。分类变量采用百分数法分析，连续变量采用中位数法分析。生存分析采用*Kaplan-Meier*法。

## 2 结果

### 2.1 患者客观疗效与生存情况

末次随访时间为2015年6月30日。中位随访时间为28.9个月，全组患者中16例死亡，16例存活，28例进展，4例未进展。

全组32例患者的中位生存时间（median OS, mOS）和中位无进展生存时间（median PFS, mPFS）分别为24.7个月（4.5个月-51.6个月）和11.2个月（1.0个月-31.4个月），RR和DCR分别为62.5%和93.8%。

吉非替尼初治患者的mOS和mPFS分别为35.6个月（8.6个月-51.6个月）和11.3个月（3.3个月-31.4个月），RR和DCR分别为75.0%和100.0%。吉非替尼复治患者的mOS和mPFS分别为18.6个月（4.7个月-26.0个月）和6.7个月（1.0个月-29.7个月），RR和DCR分别为50.0%和83.3%（图 1A和图 1B）。

**1:**
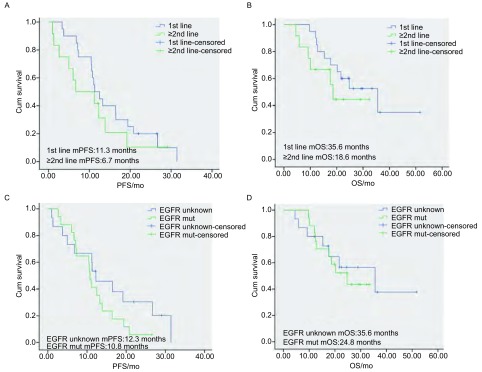
患者的各种生存曲线。A：初治及复治患者的无进展生存时间；B：初治及复治患者的总生存时间；C：*EGFR*敏感性突变与突变状态不明患者的无进展生存时间；D：*EGFR*敏感性突变与突变状态不明患者的总生存时间 Survival curves of patients. A: Progression-free survival (PFS) of untreated patients and pretreated patients; B: Overall survival (OS) of untreated patients and pretreated patients; C: PFS of epithelial growth factor receptor (*EGFR*)-mutated patients and EGFR-unknown patients; D: OS of *EGFR*-mutated patients and EGFR-unknown patients

*EGFR*敏感性突变患者的mOS和mPFS分别为24.8个月（4.5个月-51.6个月）和10.8个月（3.1个月-26.4个月），RR和DCR分别为75.0%和100.0%。*EGFR*突变状态不明患者的mOS和mPFS分别为35.6个月（3.3个月-25.6个月）和12.3个月（1.0个月-31.4个月），RR和DCR分别为53.3%和86.7%（图 1C和图 1D）。

### 2.2 患者不良反应发生情况

全组患者耐受性好，未观察到3级-4级不良事件。常见的1级-2级不良事件包括皮疹15例（46.9%）、腹泻7例（21.9%）、口腔溃疡1例（3.1%）。

## 3 讨论

EGFR是ErbB（erythroblastosis oncogene B）/HER（human epidermal growth factor receptor）家族成员之一。EGFR激活后，磷酸化形成二聚体，激活下游Ras-Raf-MEK和PI3K/Akt这两条信号通路，控制细胞的增殖、分化、凋亡、侵袭以及血管形成^[7, 8]^。目前认为，EGFR在肿瘤细胞中的异常激活通常有以下三种机制：①非配体依赖型EGFR的过表达；②编码EGFR酪氨酸激酶激活结构域的基因突变，包括点突变和缺失突变；③肿瘤细胞通过自分泌作用过表达转化生长因子（transforming growth factor, TGF）-α，与EGFR结合后激活下游信号通路^[7, 10-12]^。临床前研究^[13]^证明EGFR-TKI对于NSCLC脑转移有效。后续临床研究^[14-16]^报道吉非替尼治疗对于NSCLC脑转移的有效率在10%-38%之间，中位持续应答时间在9个月-13.5个月，亦有数项临床研究^[17-19]^证实厄洛替尼对于NSCLC治疗脑转移有效，这些临床研究结果显示EGFR-TKI治疗NSCLC脑转移的客观疗效和PFS与颅外病灶相当。

本研究中，全组患者的RR和DCR高达62.5%和93.8%，在*EGFR*突变状态不明的亚组人群中RR和DCR仍然高达53.3%和86.7%。究其原因，有以下两点：①入组人群中有65.6%患者接受过脑转移灶局部放疗；②有50%的患者为*EGFR*敏感性突变，余50%患者虽然*EGFR*突变状态不明，但其中绝大多数患者是既往无吸烟史的老年女性。但值得注意的是，既往研究^[20, 21]^提示对于有*EGFR*敏感性突变的NSCLC脑转移患者，应用EGFR-TKI治疗，较EGFR野生型的患者，RR更高，生存期更长。一项对69例患者的回顾性研究^[21]^结果表明对于有*EGFR*基因突变的患者和突变状态不明的患者接受厄洛替尼治疗其PFS分别为11.7个月和5.8个月（*P* < 0.05），OS分别为12.9个月和3.1个月（*P* < 0.001）。Kim等^[22]^报道了对于初治的患者，EGFR-TKI对于中枢神经系统病灶的有效率高达70%。在本研究中初治和复治患者的有效率分别为70.0%和50.0%。

既往研究^[23]^已经证明，*EGFR*敏感性突变的人群对于EGFR-TKI的疗效优于*EGFR*突变状态未明患者。但是在该研究中EGFR状态不明患者的生存优于敏感突变患者，这与该研究纳入人群数量较少，*EGFR*突变阳性组患者的基线状态差于EGFR状态不明组等因素相关。

在不良反应方面，该研究提示吉非替尼安全性较好。全组患者未观察到肝功能损伤，仅有轻度的皮疹15例（46.9%）、腹泻7例（21.9%）、口腔溃疡1例（3.1%）。本组研究中观察到皮疹出现者疗效优于未出皮疹者。

临床上，一代EGFR-TKI吉非替尼已经广泛应用于晚期NSCLC的治疗。但对于EGFR-TKI在NSCLC脑转移患者的治疗，临床数据相对欠缺，一系列问题亟待探索，如对于有症状或无症状NSCLC脑转移患者的一线选择；吉非替尼与脑转移灶局部放疗是否应该联合应用亦或是序贯应用等。该研究为回顾性研究，样本量较少，虽然我们观察到吉非替尼治疗NSCLC脑转移患者有效率较高且耐受性好，但尚需多中心随机临床试验的结果来验证。
